# A Winter in the Azores, and a Summer at the Baths of the Furnas

**Published:** 1841-07

**Authors:** 


ART. VI.—*A Winter in the Azores, and a Summer at the Baths of the Furnas.* By JOSEPH BULLAR, M.D., and HENRY BULLAR, of Lincoln's Inn.—*London*, 1841. Two vols. 8vo, pp. 375-390. With two coloured Lithographs and numerous Woodcuts.

WE opened these volumes solely with the view of seeing what the authors had to say of the remedial qualities of the mineral waters of the Azores, and the sanative influence of their climate. We must confess, however, that we did not part with the work until we had perused the whole of it, so cleverly and beautifully is it written, and so full of interesting details respecting places and people, of which and of whom so very little is known in this country. It has not been our fate for many a day to read so amusing and so pleasant a book of travels.

The mineral waters in the valley of the Furnas, in St. Michael's, are of five different kinds: a boiling alkaline water; a hot carbonated water; a mixture of hot alkaline water and cold carbonated chalybeate water; a highly carbonated cold chalybeate water; a tepid carbonated water. The chemical analysis and medicinal uses of these waters are given in an appendix, as is also a complete view of the climate of the islands. This last is singularly equable and mild, and cannot fail to be useful in certain forms of pulmonary and other chronic diseases. The hot baths are also well calculated to benefit persons affected with gout, rheumatism, skin diseases, &c., and they seem admirably suited to reduce the morbidly corpulent to more wieldy dimensions. They are in considerable requisition for this purpose with the natives, who seem to rival our very aldermen in this civic distinction. We cannot resist the temptation of extracting a few paragraphs on this head, which, in sportive language, contain much important truth, and afford a specimen of the charming stile in which these volumes are composed:

"The women of all the classes above the poor keep very much at home; the men of the same ranks are neither active sportsmen nor pedestrians, nor do they indulge in any of the manly games of our own countrymen, or (as far as I

have seen) in any substitute for them. Those in trade conduct it in the most leisurely way, shutting up their shops at two o'clock; and they are as scrupulous in attending to the red-letter days in the almanack as an English schoolboy used to be when such events were more observed than they are now. Few ride on horseback; the majority move along upon the backs of easy-paced asses, sitting sideways on a soft cushion. The inevitable consequence follows. Man's body is alone suited to earning his bread by the sweat of his brow, and he cannot defy that curse without a heavy punishment. If he need not earn his bread himself, he must substitute laborious pleasures: he must work harder than a post-boy, under the name of hunting; or for mere relaxation, encounter such cold and wet, hunger, thirst, and fatigue in deer-stalking or grouse-shooting, as a half-famished North American Indian meets with who has a starving family depending on his success; or he must rise early and work harder than a labourer in toiling over ploughed ground and stubble fields, or through wet turnips and thick grass in pursuit of partridges or hares; or he must walk up and down the same street in the same country town with all the assiduity of a policeman, to the market, or his club, or newsroom: or with his wife, or for his wife; or he must play at bowls or cricket, at gardening or at navigation; if he does none of these things or similar ones, he grows fat, has indigestion, and consults doctors with the vain hope of being enabled to baffle nature with impunity for some little time longer, and after a few years of perpetual uneasy feelings, it is found that his heart is diseased, he becomes dropsical, or loses the use of one half of his body, and is wheeled about in a chair, imbecile in mind as well as in limbs, or he becomes melancholy, and suspicious of his best friends, or by some such winding-up he arrives at the last scene that ends his common-place eventless history.

"Even to an English eye, accustomed as it is to 'The fair round belly with good capon lined,' the prodigality of fat Azoreans is striking. It is generally thought that England has a monopoly of human fat; that the conditions favourable to its inordinate growth, such as easy circumstances, abundance of beef and mutton from rich pastures, duly moistened with strong beers or strong wines, together with a constitutional selfish quietude of disposition and capacious hereditary powers of digestion,—the combined result of many generations of generous livers,—are alone found under our constitution. The Scotch have a 'lean and hungry look, they think too much;' the Irish are too excitable or too poor; the French have, it is true, enormous appetites, but how can they fatten on bread, beans, dried peas, and thin wine? You may travel through their countries and not meet with more exceptions than are enough to prove the rule. I should doubt the existence of much fat in America; the unexampled busymindedness there must prevent all such accumulations. But here the necessary requisites are found; the rich viands and the beer are wanting, but the climate is superior; no extremes; no cold driving the fat man to unusual exertion, nor that other extreme, intense heat, melting him into a finer form; but throughout the year, by night and by day, an equable greenhouse warmth, keeping the body, even when passive, in a genial glow, and enticing it to quietude and repose.

"The Castle of Indolence might have been built here; and he, who when smitten with the delights of leisure, declared, that if he had a son he should do nothing, and be called Nothing-to-do, should have transported him to this island. No speculations are there to vex the genial current of the soul of the indolent man, who would be richer without labouring for it: politics, instead of a daily excitement, are a monthly or quarterly one, softened by time and distance; there is no literature to set men thinking,—midnight-oil is never burnt,—a face sicklied o'er with the pale cast of thought is not, and no foolish, over-careful Azoreans break their sleep with thoughts, their brains with care, their bones with industry.

"They say that men, who when they arrive at the Furnas look like huge hills of flesh, after soaking an hour a day in the very hot water, and encouraging dissolution and thaw, for an hour or two afterwards, by lying upon a board covered with thick woollen cloaks, with towels wound round their heads and necks, return so slim as to be hardly recognized by their nearest friends: the baths using up their spare materials as a winter's starvation does those of a hybernating dormouse. There are now a few portly individuals, sleek-headed men, and such as sleep o' nights, who seem to be here for this reducing process. As a remedy against obesity these baths may be highly useful, for they are means likely to be employed, as they require no self-denial. Order a sensual man to take hard exercise, little sleep, and less food, and you are sure to be unattended to; but direct him to use a luxury, and he may, in following his old habits, take the advice." (pp. 190-96.)

